# The Impact of Early Life Stress on Anxiety Symptoms in Late Adulthood

**DOI:** 10.1038/s41598-019-40698-0

**Published:** 2019-03-13

**Authors:** Anna Lähdepuro, Katri Savolainen, Marius Lahti-Pulkkinen, Johan G. Eriksson, Jari Lahti, Soile Tuovinen, Eero Kajantie, Anu-Katriina Pesonen, Kati Heinonen, Katri Räikkönen

**Affiliations:** 1 Department of Psychology and Logopedics, Faculty of Medicine, University of Helsinki, Helsinki, Finland; 2 Queen’s Medical Research Institute, University of Edinburgh, Edinburgh, United Kingdom; 3 Department of General Practice and Primary Health Care, University of Helsinki and Helsinki University Hospital, Helsinki, Finland; 4 Chronic Disease Prevention Unit, Department of Public Health Solutions, National Institute for Health and Welfare, Helsinki, Finland; 5 Folkhälsan Research Center, Helsinki, Finland; 6 Children’s Hospital, Helsinki University Hospital and University of Helsinki, Helsinki, Finland; 7 PEDEGO Research Unit, MRC Oulu, Oulu University Hospital and University of Oulu, Oulu, Finland

## Abstract

Early life stress (ELS) may increase the risk of anxiety throughout the life course. Whether this effect extends to late adulthood is poorly known. In our study comprising 1872 participants from the Helsinki Birth Cohort Study born in 1934–1944, we investigated the association of various forms of ELS and their accumulation with self-reported anxiety symptoms at the age of 65–77 years. Data on childhood socioeconomic status and separation from parents were based on national registers for all participants. Information on self-reported emotional and physical trauma, parental divorce, and death of a family member in childhood was obtained from 1277 participants. We found that experiencing emotional trauma, physical trauma, and low socioeconomic status in childhood were associated with increased anxiety symptoms in late adulthood [B = 0.44 (95% CI = 0.31–0.58); B = 0.33 (95% CI = 0.20–0.46); B = 0.10 (95% CI = 0.01–0.19), respectively]. These associations remained significant even after controlling for other forms of ELS. Accumulation of early life stress also increased the levels of late-adulthood anxiety symptoms and the risk of anxiety regarded as clinically significant. Screening for potentially stressful childhood experiences in elderly populations may help identifying individuals with increased anxiety symptoms and planning preventive and therapeutic interventions for those exposed to ELS.

## Introduction

Anxiety is a common, yet a poorly detected psychiatric problem in late adulthood^1,2^. Subclinical anxiety symptoms are even more frequent than diagnosed anxiety disorders^1,3^. Anxiety has a wide-ranging negative impact on elderly adults’ functioning: it is associated with lower cognitive performance, memory and sleeping disturbances, and a higher risk of somatic illnesses^4–7^. However, the developmental risk factors for late-life anxiety are not well understood.

One potential risk factor for late-life anxiety is early life stress (ELS), such as exposure in childhood to maltreatment, separation from parents or parental divorce, death of a family member, and poverty. According to several studies^8–14^ and meta-analyses^15–17^, ELS exposure is associated with an increased risk of adulthood anxiety as well as a number of other psychiatric problems, including depression^17,18^, substance use disorders^17,19^, personality disorders^20,21^, psychosis^22^ and suicidality^17,23^. These associations have been suggested to be mainly unspecific, with different types of ELS increasing the risk of different types of psychiatric outcomes in a similar but consistent manner^17,24^. Since the association of ELS with particularly late-adulthood anxiety is not known well regardless of the relatively high prevalence of anxiety symptoms in elderly populations^1,2^, we chose this as the focus of the current study.

Research comparing the role of different ELS types in late-life anxiety is sparse^25–28^. Still, the mechanisms linking ELS to later anxiety seem to persist throughout the lifespan^29–33^. Also, the accumulation of early life adversities is suggested to increase the anxiety risk still in late adulthood^26^. It is challenging to assess which ELS forms have the strongest association with later anxiety, since their co-occurrence is high^34^. It has been suggested, however, that especially interpersonal trauma, e.g. physical or emotional abuse, has a stronger association with brain structure alterations increasing psychopathology risk compared to other ELS types such as parental divorce or death of a relative^35^. There is also evidence that childhood emotional abuse may increase later anxiety risk more than physical abuse does^17^.

Previous findings on the associations between separation from parents due to illness, death, divorce etc. and anxiety are inconsistent and scarce among the elderly. Although no coherent association has been found between parental divorce and later anxiety^36,37^, more research is needed^36^. Also, no consistent association has been found between separation from parents and later anxiety^19,38^, although some evidence suggests that parental separation is a stronger risk factor for anxiety and other psychopathology than parental loss is^11,39^.

In this study, we examined the relationship of various forms of potentially stressful childhood experiences referred to as ELS – namely emotional and physical trauma, low socioeconomic status (SES), separation from parents, death of a family member, and parental divorce – with late adulthood anxiety symptoms. We investigated both the effects of independently occurring ELS experiences and of their accumulation. Based on earlier studies, we hypothesized that ELS experiences, especially emotional trauma, and their accumulation are associated with higher anxiety symptoms in late adulthood.

## Materials and Methods

### Participants

Our study sample is a part of the Helsinki Birth Cohort Study (HBCS), comprising all 13345 individuals (52.3% male) born singleton in one of the two maternity hospitals in Helsinki, Finland, between 1934 and 1944. The HBCS, described in detail elsewhere^40^, has been approved by the Ethics Committee of the National Public Health Institute and was carried out in accordance with the relevant guidelines and regulations. In 2009–2010, a battery of psychological questionnaires measuring e.g. anxiety symptoms was delivered to 4147 randomly selected HBCS participants, then aged 65–77 years. Of them, 2935 were reached^41^, and 1893 (64.5%) returned the questionnaire. All participants gave their informed consents to participate. Our final sample comprised 1872 (63.8%) participants (1082 male) completing the anxiety questionnaire. Those who returned the questionnaire answering less than half of the anxiety symptom questions, being thus excluded (n = 21), did not differ from the study sample regarding age, sex, or ELS exposure (p ≥ 0.30).

### Anxiety symptoms in late adulthood

Late adulthood anxiety symptoms were assessed with the Finnish version of the Beck Anxiety Inventory (BAI)^42^ that comprises 21 statements describing anxiety symptoms. The participants report the frequency of each symptom during the past week on a scale from 0 to 3. Therefore, the BAI scores vary from 0 to 63. Higher scores reflect more severe anxiety symptoms. For participants leaving less than half of the BAI questions unanswered (n = 69), missing values were replaced with the mean of the participant’s answers. A BAI cutoff score of 16 has been suggested to refer to clinically significant anxiety symptoms, described as “moderate” or “severe”^43,44^. We used this cutoff score to further investigate the association between ELS and clinically significant anxiety.

The BAI has good reliability^42,43^ and convergent validity^42,45^. The validity is good also among the elderly^46^. In this study, the internal consistency of the BAI was α = 0.86.

### Separation from parents

During World War II, approximately 80000 Finnish children were evacuated without their parents due to wartime insecurity. Many were sent unaccompanied to Sweden and Denmark, and siblings were often placed in different families^47^. We extracted data on these evacuations, age at first separation, and duration of separations from Finnish National Archives registers. Data on age at first separation was available for 194 of the 210 evacuated participants. In our study sample, the mean separation age was 4.42 years (SD 2.33, range 0–10 years). The mean separation duration was 1.76 years (SD 1.05, range 0–7 years).

140 participants reported having been evacuated during the war although no register data on the evacuation were found. These participants may have been evacuated unofficially, e.g. by staying with relatives. In our analyses, participants with only self-reported separation were coded to a separate group, while participants with both self-reported and register-based separation were coded to the category of register-based separation.

### Low childhood SES

A dichotomous variable of childhood SES was defined by the highest recorded occupational status (manual/clerical worker) of either parent. Data on parents’ occupational status were extracted from birth-, child welfare clinic-, and school healthcare records, and was available for 1871 participants.

### Self-reported traumatic experiences

Traumatic experiences in childhood were assessed retrospectively with the Traumatic Experiences Checklist (TEC)^48^. TEC is a self-report questionnaire that comprises statements describing potentially traumatic life events. The questionnaire has good psychometric properties^48,49^. The TEC questionnaire was sent to a subsample of the larger cohort of 13345 participants. Of our study sample of 1872 participants completing the BAI, 1277 had completed the TEC questionnaire in 2001–2004, at age 57–70 years. The mean time difference between completing the TEC questionnaire and the BAI questionnaire was 5.68 years (SD 0.45, range 4.66–6.37 years). Within the TEC statements there are six specific statements measuring physical trauma and six measuring emotional trauma (e.g. “Physical violence (e.g. hitting, injuring) by a parent and/or a sibling” and “Unexpected or unreasonable punishment” for physical trauma and “Emotional violence (e.g. bullying, calling names, invalidating, verbal threatening, unjust punishment) by a parent and/or a sibling” and “Indifference towards my needs (e.g. insufficient tenderness, being left alone) by a parent and/or a sibling” for emotional trauma). These twelve statements were used in our study. Binary variables on the occurrence of physical and emotional trauma were formed, “yes” indicating that the participant had experienced at least one of the six physical or emotional potentially traumatic events. Altogether 1040 participants replied to the questions regarding physical trauma and 1023 participants to those regarding emotional trauma. The participants also reported the age at the event.

In our analyses, participants reporting no physical or emotional trauma or trauma only in adulthood were used as a comparison group. Participants with missing values either in the question on the traumatic experience or on its age were excluded from the analyses regarding physical or emotional trauma.

### Parental divorce

Of the participants, 1213 answered the TEC statement “My parent’s divorce” on parental divorce in childhood and age at divorce. Participants reporting no parental divorce or parental divorce only in adulthood formed a comparison group.

### Death of a family member

Of the participants, 1195 answered the TEC statement “Death of a family member in childhood or adolescence (brother, sister, parent)” on the death of a family member and age at death of a family member. Participants reporting no death in the family or death of a family member only in adulthood formed a comparison group.

### Covariates

Sex and age at completing the BAI questionnaire were used as covariates. Participants’ date of birth and sex were extracted from birth records. Age of the participants was calculated by subtracting birth date from the BAI completion date. In the analyses regarding ELS experiences measured with the TEC questionnaire (physical and emotional trauma, parental divorce and death of a family member), also the time interval between completing the TEC questionnaire and the BAI questionnaire was used as a covariate.

To examine whether adulthood SES mediated the association between ELS and late-life anxiety, we also conducted additional analyses using adulthood SES as a covariate. Adulthood SES was based on the participant’s own highest level of education achieved. Information on adulthood SES was available for all 1872 participants. Low adulthood SES was defined as an education level of upper secondary school or lower (n = 1060).

### Statistical analyses

A square root conversion and standardization to standard deviation units were made for the BAI scale to correct for skewness and to facilitate effect size interpretation. The associations between covariates and anxiety were examined using independent samples t-tests and Pearson’s correlation analyses. The number of participants included in the analyses for each type of ELS, including the types of ELS measured with the TEC, is presented in Table 1. Linear regression analyses were used to investigate the associations of self-reported physical and emotional traumas, low childhood SES, separation from parents, parental divorce, and death of a family member, the accumulation of these stressors, and age at ELS, with anxiety symptoms. Separate models were used to investigate the individual associations of each ELS type with anxiety (Model 1) and the independence of these associations from other ELS types (Model 2). In Model 1, each ELS type was investigated separately as an independent variable, controlling for sex, age and the time difference between the TEC and the BAI as covariates. In Model 2, all ELS types were entered to the analyses simultaneously, also controlling for sex, age and the time interval between the TEC and the BAI. Participants with missing values regarding one ELS variable were not excluded from analyses regarding all other ELS variables. This was done by encoding missing values of each ELS type to a separate category. Since different types of ELS were highly inter-correlated, no adjustments for multiple comparisons were used in the analyses since for example, the independent test assumptions of the Bonferroni corrections were not met.

**Table 1:** Subsamples of the study sample (n = 1872) regarding different analyses.

	N
BAI	1872
Childhood SES	1871
Separation from parents	1872
TEC	1277
TEC Physical trauma	1040
TEC Emotional trauma	1023
TEC Parental divorce	1213
TEC Death of a family member	1195

To examine the accumulation of different types of ELS, an ordinal variable of 0, 1, 2, or 3 or more separate ELS types was used for linear regression analyses. Since there were much fewer participants reporting 4, 5, or 6 accumulated ELS experiences than participants reporting three or less ELS experiences (Table 2), participants reporting three or more ELS experience types were combined to a single group in order to achieve comparable group sizes and to improve the reliability of the statistical analyses. Participants with more than three missing values on stressful experiences were excluded from the accumulation analyses.

**Table 2:** Characteristics of the study population.

	N	mean (SD)/N (%)
Age at follow up	1872	68.73 (2.84)
Beck Anxiety Inventory score^a^	1872	6.31 (5.84)
Physical trauma experienced in childhood (Yes)^b^	1040	278 (26.70)
The number of physically traumatic experiences in childhood		0.63 (1.07)
0		572 (67.30)
1		113 (13.30)
2		96 (11.30)
3 or more		69 (8.10)
Age at first physically traumatic experience	278	
Less than 5 years		35 (12.60)
5–12 years		179 (64.40)
12–18 years		64 (23.00)
Emotional trauma experienced in childhood (Yes)^b^	1023	299 (29.20)
The number of emotionally traumatic experiences in childhood		0.66 (1.19)
0		651 (68.50)
1		125 (13.20)
2		81 (8.50)
3 or more		93 (9.80)
Age at first emotionally traumatic experience	299	
Less than 5 years		65 (21.70)
5–12 years		166 (55.50)
12–18 years		68 (22.70)
Childhood SES^c^	1871	
Manual worker		796 (42.50)
Clerical worker		1075 (57.50)
Separation from parents in childhood	1872	
Evacuated		210 (11.20)
Self-reported evacuation		140 (7.50)
Not evacuated		1522 (81.30)
Age at evacuation (registered evacuees)	194	4.42 (2.33)
Parental divorce in childhood (Yes)^b^	1213	97 (8.00)
Age at parental divorce in childhood (years)		8.64 (4.94)
Death of a family member in childhood (Yes)^b^	1195	186 (15.60)
Age at death of a family member in childhood (years)		8.73 (4.85)
Accumulation of stressful life experiences	1266	
0 stressful life experiences		309 (24.40)
1 ELS type		472 (37.30)
2 ELS types		292 (23.10)
3 ELS types		130 (10.30)
4 ELS types		54 (4.30)
5 ELS types		8 (0.60)
6 ELS types		1 (0.10)

^a^The original BAI scale score.

^b^The participants reporting no trauma or trauma only in adulthood were used as a control group.

^c^Childhood SES is defined by the highest recorded occupational status of either the father or the mother of the participant in childhood.

With logistic regression analysis, we further examined the association of each ELS type and their accumulation with anxiety symptoms above the clinically significant symptom cutoff score. Models 1 and 2 with the abovementioned independent variables and covariates were used also here. Furthermore, additional linear and logistic regression analyses were conducted using adulthood SES as a covariate.

Finally, since some studies suggest that sex^35,50^, age at ELS exposure^11,21^, and childhood SES^19^ may moderate the associations between ELS and psychopathology risk, we studied the effects of age at ELS and interactions of sex and SES with ELS using linear regression analysis, with sex and follow-up age as covariates.

## Results

### Characteristics of the study sample

The sample characteristics are presented in Table 2. The participants’ mean age at follow-up was 68.73 years (SD = 2.84, range 65–77). The mean BAI score was 6.31 (SD 5.84, range 0–42). Women [mean difference 2.02 (95% CI 1.48–2.56), p < 0.001] and older participants [r = 0.14, p < 0.001] had higher anxiety symptoms. The different childhood stressors co-occurred often (Table 3).

**Table 3:** The concurrent occurrence of ELS types.

	Self-reported emotional trauma		Low childhood SES		Separation from parents			Parental divorce		Death of a family member	
	N (%^a^)	χ^2^	N (%^a^)	χ^2^	Register-based N (%^a^)	Self-report only N (%^a^)	χ^2^	N (%^a^)	χ^2^	N (%^a^)	χ^2^
Self-reported physical trauma											
Yes	174 (66.40)	218.01***	108 (38.80)	3.56	36 (12.90)	44 (15.80)	12.97**	33 (12.40)	5.50*	38 (14.00)	1.24
No	118 (17.10)		346 (45.40)		75 (9.80)	69 (9.10)		56 (7.60)		124 (16.90)	
Self-reported emotional trauma											
Yes			119 (39.80)	3.59	42 (14.00)	48 (16.10)	18.36***	41 (14.60)	18.03***	59 (20.30)	4.41*
No			335 (46.30)		66 (9.10)	65 (9.00)		44 (6.20)		103 (14.80)	
Low childhood SES											
Yes					105 (13.20)	62 (7.80)	5.83	40 (7.50)	0.35	90 (17.40)	2.28
No					105 (9.80)	78 (7.30)		57 (8.40)		96 (14.20)	
Separation from parents											
Register-based								9 (6.50)	7.09*	27 (20.00)	6.21*
Self-report only								18 (14.00)		27 (20.90)	
No separation								70 (7.40)		132 (14.20)	
Parental divorce											
Yes										14 (14.40)	0.14
No										167 (15.90)	

^a^The percentage of those reporting ‘yes’ to the ELS type shown on the row.

*p < 0.05 **p < 0.01 ***p < 0.001.

### ELS and anxiety symptoms in late adulthood

Table 4 shows that emotional and physical traumas and low childhood SES were each associated with higher anxiety symptoms in late adulthood (Model 1; Table 4). Also, when all stressors were added to the analyses simultaneously (Model 2), emotional and physical traumas and low childhood SES remained associated with higher anxiety symptoms independently of other stressors (Table 4).

**Table 4:** Linear regression analyses of the association of potentially stressful events in childhood with anxiety symptoms in late adulthood.

	N	B	95% CI		p
Self-reported physical trauma in childhood(yes/no)					
Model 1^a^	1040	0.33	0.20	0.46	<0.001
Model 2^b^	1040	0.17	0.01	0.32	0.03
Self-reported emotional trauma in childhood (yes/no)					
Model 1^a^	1023	0.44	0.31	0.58	<0.001
Model 2^b^	1023	0.37	0.22	0.53	<0.001
Childhood SES (manual worker/clerical worker)					
Model 1^a^	1871	0.10	0.01	0.19	0.02
Model 2^b^	1871	0.12	0.03	0.21	0.01
Separation from parents					
Evacuee - Model 1^a^	1872	0.08	−0.07	0.23	0.31
Evacuee - Model 2^b^	1871	0.02	−0.13	0.17	0.81
Self-reported separation - Model 1^a^	1872	0.14	−0.03	0.32	0.11
Self-reported separation - Model 2^b^	1871	0.08	−0.10	0.26	0.38
Parental divorce					
Model 1^a^	1213	0.16	−0.05	0.36	0.13
Model 2^b^	1213	0.09	−0.11	0.30	0.36
Death of a family member					
Model 1^a^	1195	0.06	−0.10	0.21	0.46
Model 2^b^	1195	0.04	−0.12	0.19	0.63
Accumulation of risk factors^c^					
1 stressful event	1266	0.19	0.05	0.33	0.01
2 stressful events	1266	0.33	0.17	0.48	<0.001
3 or more stressful events	1266	0.46	0.28	0.63	<0.001

^a^The studied stressful experience was included in the model as an independent variable, while age and sex were used as covariates. For physical trauma, emotional trauma, parental divorce and death of a family member, also the interval between TEC and BAI was used as a covariate.

^b^The studied stressful experience was included in the model as an independent variable with other stressful experiences, while age and sex were used as covariates. For physical trauma, emotional trauma, parental divorce and death of a family member, also the interval between TEC and BAI was used as a covariate.

^c^The person has experienced 0, 1, 2, or 3 or more different ELS types in childhood. The group with 0 stressful events in childhood functions as a comparison group.

Emotional and physical trauma and parental divorce were also each individually associated with an increased risk for anxiety symptoms exceeding the clinical cutoff score (Model 1; Table 5). In Model 2, the increased risk of clinically significant anxiety symptoms remained significant for emotional trauma, parental divorce and also marginally for low childhood SES but not for physical trauma (Table 5).

**Table 5:** Logistic regression analyses of the association between potentially stressful events in childhood and the odds ratio of clinically significant anxiety symptoms in late adulthood.

	N	OR	95% CI		p
Self-reported physical trauma in childhood(yes/no)	1040				
Model 1^a^		1.73	1.10	2.74	0.02
Model 2^b^		1.04	0.61	1.78	0.88
Self-reported emotional trauma in childhood (yes/no)	1023				
Model 1^a^		2.78	1.78	4.33	<0.001
Model 2^b^		2.59	1.53	4.39	<0.001
Childhood SES (manual worker/clerical worker)	1871				
Model 1^a^		1.36	0.98	1.91	0.07
Model 2^b^		1.42	1.01	2.00	0.05
Separation from parents					
Evacuee - Model 1^a^	1872	1.44	0.88	2.35	0.14
Evacuee - Model 2^b^	1871	1.24	0.75	2.05	0.40
Self-reported separation - Model 1^a^	1872	1.26	0.71	2.25	0.44
Self-reported separation - Model 2^b^	1871	1.01	0.55	1.83	0.99
Parental divorce	1213				
Model 1^a^		2.37	1.33	4.19	0.003
Model 2^b^		2.14	1.18	3.88	0.01
Death of a family member	1195				
Model 1^a^		1.09	0.64	1.84	0.75
Model 2^b^		1.03	0.60	1.77	0.92
Accumulation of risk factors^c^	1266				
1 stressful event		1.43	0.79	2.59	0.24
2 stressful events		1.94	1.04	3.60	0.04
3 or more stressful events		2.91	1.55	5.46	0.001

^a^The studied stressful experience was included in the model as an independent variable, while age and sex were used as covariates. For physical trauma, emotional trauma, parental divorce and death of a family member, also the interval between TEC and BAI was used as a covariate.

^b^The studied stressful experience was included in the model as an independent variable with other stressful experiences, while age and sex were used as covariates. For physical trauma, emotional trauma, parental divorce and death of a family member, also the interval between TEC and BAI was used as a covariate.

^c^The person has experienced 0, 1, 2, or 3 or more stressful events in childhood. The group with 0 stressful events in childhood functions as a comparison group.

Separation from parents and death of a family member were not significantly associated with either dimensional or categorical anxiety symptoms (Tables 4 and 5).

### Accumulation of ELS

**Figure 1:**
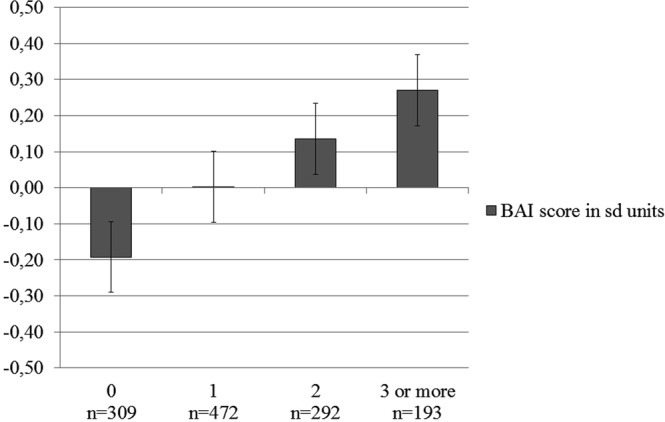
Accumulation of different types of ELS. The association between the accumulation of different ELS types and the standardized residual of the BAI score [in standard deviation (sd) units] after adjusting for age and sex as covariates. The accumulation of ELS is defined by the number of different stressful experience types in childhood (0−3 or more). Error bars represent the 95% confidence interval.

Our ELS accumulation analyses showed that independently of age and sex, a higher number of different types of ELS experiences was associated with higher anxiety symptoms (Table 4; Fig. 1). This was found for both dimensional and categorical anxiety symptoms. Encountering one potentially stressful childhood experience did not significantly increase the likelihood of anxiety symptoms exceeding the clinical cutoff, whereas the accumulation of two or more ELS types did (Table 5).

### The effect of adulthood SES on the association between ELS and late-life anxiety

Low adulthood SES did not have an effect on the associations between different ELS types and anxiety symptoms except for the association between low childhood SES and later anxiety, which was no longer significant for either the continuous or the categorical anxiety symptoms (Tables 6 and 7). Also, the accumulation of two ELS types was only marginally associated with clinically significant anxiety symptoms. In contrast, the effects of emotional and physical trauma, parental divorce and accumulation of ELS regarding continuous anxiety symptoms were all independent of adulthood SES.

**Table 6:** Linear regression analyses of the association of potentially stressful events in childhood with anxiety symptoms in late adulthood with adulthood SES included in the models.

	N	B	95% CI		p
Self-reported physical trauma in childhood(yes/no)	1040				
Model 1^a^		0.34	0.21	0.47	<0.001
Model 2^b^		0.18	0.03	0.32	0.02
Self-reported emotional trauma in childhood (yes/no)	1023				
Model 1^a^		0.45	0.32	0.58	<0.001
Model 2^b^		0.38	0.22	0.53	<0.001
Childhood SES (manual worker/clerical worker)	1871				
Model 1^a^		0.03	−0.06	0.12	0.48
Model 2^b^		0.05	−0.04	0.14	0.27
Separation from parents					
Evacuee - Model 1^a^	1872	0.05	−0.10	0.19	0.55
Evacuee - Model 2^b^	1871	−0.01	−0.15	0.14	0.90
Self-reported separation - Model 1^a^	1872	0.12	−0.06	0.29	0.18
Self-reported separation - Model 2^b^	1871	0.05	−0.12	0.23	0.55
Parental divorce	1213				
Model 1^a^		0.12	−0.08	0.32	0.24
Model 2		0.05	−0.15	0.25	0.60
Death of a family member	1195				
Model 1^a^		0.05	−0.11	0.20	0.56
Model 2^b^		0.03	−0.12	0.18	0.71
Accumulation of risk factors	1266				
1 stressful event		0.16	0.03	0.30	0.02
2 stressful events		0.31	0.15	0.46	<0.001
3 or more stressful events		0.39	0.22	0.57	<0.001

^a^The studied stressful experience was included in the model as an independent variable, while age, sex and adulthood SES were used as covariates. For physical trauma, emotional trauma, parental divorce and death of a family member, also the interval between TEC and BAI was used as a covariate.

^b^The studied stressful experience was included in the model as an independent variable with other stressful experiences, while age, sex and adulthood SES were used as covariates. For physical trauma, emotional trauma, parental divorce and death of a family member, also the interval between TEC and BAI was used as a covariate.

^c^The person has experienced 0, 1, 2, or 3 or more different ELS types in childhood. The group with 0 stressful events in childhood functions as a comparison group.

**Table 7:** Logistic regression analyses of the association between potentially stressful events in childhood and the odds ratio of clinically significant anxiety symptoms in late adulthood with adulthood SES included in the models.

	N	OR	95% CI		p
Self-reported physical trauma in childhood(yes/no)	1040				
Model 1^a^		1.80	1.13	2.87	0.01
Model 2^b^		1.05	0.61	1.82	0.87
Self-reported emotional trauma in childhood (yes/no)	1023				
Model 1^a^		2.94	1.86	4.64	<0.001
Model 2^b^		2.77	1.60	4.78	<0.001
Childhood SES (manual worker/clerical worker)	1871				
Model 1^a^		1.14	0.81	1.60	0.46
Model 2^b^		1.19	0.84	1.69	0.34
Separation from parents					
Evacuee - Model 1^a^	1872	1.34	0.82	2.19	0.25
Evacuee - Model 2^b^	1871	1.16	0.70	1.93	0.56
Self-reported separation - Model 1^a^	1872	1.19	0.67	2.14	0.55
Self-reported separation - Model 2^b^	1871	0.92	0.51	1.69	0.80
Parental divorce	1213				
Model 1^a^		2.18	1.22	3.90	0.01
Model 2^b^		1.93	1.05	3.54	0.03
Death of a family member	1195				
Model 1^a^		1.04	0.61	1.77	0.89
Model 2^b^		0.98	0.56	1.70	0.93
Accumulation of risk factors	1266				
1 stressful event		1.33	0.73	2.42	0.35
2 stressful events		1.81	0.97	3.39	0.06
3 or more stressful events		2.43	1.29	4.60	0.006

^a^The studied stressful experience was included in the model as an independent variable, while age, sex and adulthood SES were used as covariates. For physical trauma, emotional trauma, parental divorce and death of a family member, also the interval between TEC and BAI was used as a covariate.

^b^The studied stressful experience was included in the model as an independent variable with other stressful experiences, while age, sex and adulthood SES were used as covariates. For physical trauma, emotional trauma, parental divorce and death of a family member, also the interval between TEC and BAI was used as a covariate.

^c^The person has experienced 0, 1, 2, or 3 or more different ELS types in childhood. The group with 0 stressful events in childhood functions as a comparison group.

**Figure 2:**
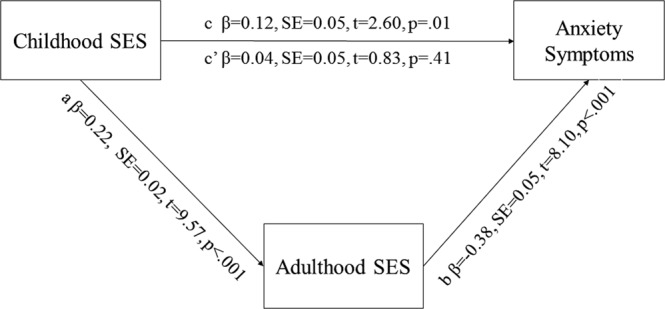
The association of childhood SES with late adulthood anxiety symptoms mediated by adulthood SES. SE represents standard errors. C represents the total effect of childhood SES on anxiety symptoms. Childhood SES no longer had a direct effect on anxiety symptoms when controlling for adulthood SES (c’). The indirect effect of childhood SES on anxiety symptoms through adulthood SES (ab) was β = 0.08 (95% CI 0.06–0.11, p < 0.001).

As further analyses, we examined whether adulthood SES mediated the effects of childhood SES on late-life anxiety. The conditions for mediation were met: childhood and adulthood SES were significantly associated with each other and both predicted higher late-life anxiety in univariate analyses, and the association between low childhood SES and late-life anxiety symptoms was no longer significant when controlling for adulthood SES. For mediation analyses, we used the bootstrapping method with 5000 resamples and bias-corrected confidence intervals, based on the *PROCESS* macro for mediation analyses by Hayes^51,52^. These analyses did not include covariates. The mediation analyses indeed showed that adulthood SES mediated the association of childhood SES and late-life anxiety symptoms (Fig. 2).

### The modifying effects of age at ELS, sex, and SES

The age at each ELS experience was not associated with anxiety symptoms (see Supplementary Table S1). Also, there were no interactions between sex and different ELS types or their accumulation (p-values ≥ 0.24) or childhood SES and other ELS types (p-values ≥ 0.22).

### The effects of missing data

To examine the effects of the data imputations on the individual BAI items, we repeated our analyses among the 1803 participants answering all 21 BAI items (see Supplementary Tables S2 and S3). The significant effects of emotional trauma, parental divorce and the accumulation of ELS on anxiety symptoms remained unchanged. However, physical trauma was now associated with increased anxiety symptoms only in Model 1, while its association with continuous anxiety symptoms in Model 2 and clinically significant anxiety symptoms in Model 1 were marginal. Also, the association of low childhood SES with continuous anxiety symptoms in Model 1 and with clinically significant anxiety symptoms was now marginal or non-significant.

## Discussion

In a large birth cohort, we compared the effects of several different forms of ELS on anxiety and found that emotional and physical childhood traumas and low childhood SES were associated with higher anxiety symptoms in late adulthood. These stressors and parental divorce were also associated with a higher risk of clinically significant anxiety symptoms. In addition, the accumulation of different ELS types was associated with higher anxiety symptoms and the risk of clinically significant anxiety.

Childhood emotional trauma and low childhood SES were most consistently associated with anxiety symptoms and the risk of clinically significant anxiety symptoms even after controlling for other ELS types, which suggests that they function as independent risk factors for anxiety. Our findings on emotional trauma support earlier studies suggesting that childhood emotional trauma may be associated with increased anxiety symptoms still in late adulthood^27,28^. Emotional trauma remains often unrecognized^53^, and yet it has been associated with several negative consequences besides anxiety, such as depression, suicidality, and drug abuse^17^, which highlights the importance of our findings. Our results further suggest that low childhood SES was independently associated with late-life anxiety symptoms regardless of other types of ELS, which contrasts previous studies that the association between childhood SES and adulthood anxiety may be explained by co-occurring stressors^54,55^. Thus, our study adds information to the scant study literature by showing that low childhood SES may carry negative consequences until old age. Low childhood SES also increases the risk of other ELS experiences^56,57^ and the risk of lower adulthood SES^58^. Our additional analyses suggest that the association between low childhood SES and late-life anxiety is mediated by a lower adulthood SES. According to our findings, the participants who had a higher SES of the childhood family had a lower risk for later anxiety symptoms, which was partly explained by them more likely attaining a higher education level in adulthood.

Childhood physical trauma was associated with higher anxiety symptoms independently of other stressors, but its association with anxiety symptoms above clinical level was no longer significant when controlling for emotional trauma. This finding is concordant with some previous studies stating that early physical trauma is associated with the risk of late-life anxiety^25,27,28^ and that the association of physical trauma with anxiety disorders may be partly explained by co-occurring ELS experiences^50,59^. Emotional trauma often occurs simultaneously with physical trauma: In our sample, 66.4% of those reporting physical trauma also reported emotional trauma (Table 3). This high co-occurrence and potential multicollinearity thereby induced to the analytic models (ϕ = 0.48, p < 0.001) may partly explain the insignificance of physical trauma as a risk factor for clinically significant anxiety symptoms when controlling for emotional trauma.

Our finding that parental divorce was not associated with continuously assessed anxiety symptoms corresponds with previous studies^36,37^. However, the finding that an independent association existed for parental divorce and clinically significant anxiety symptoms is, to our knowledge, novel. Our finding suggests that participants who had experienced parental divorce in childhood were either highly affected by the experience or not. More research is needed to further assess the role of parental divorce as a potential risk factor for late-life anxiety and to examine which factors may make an individual more vulnerable to the effects of parental divorce.

Supporting previous findings^60^, death of a family member was not associated with the risk of late-life anxiety symptoms in our study. Also our finding that parental separation due to war-time evacuation did not increase the risk for anxiety corresponds with previous findings that early separation is associated with the risk of psychopathology but not with anxiety^19,38^. Our findings regarding parental separation due to war-time evacuation cannot be generalized to all separation from parents due to special characteristics, such as the child’s worry about parents staying in war conditions, or the child being placed in a completely new environment. Also, the participants who were not separated were exposed to war-time conditions as well, which may have been a remarkable stressor as such and thus it is possible that this may partly explain the non-significance of the results. Separation from caregivers due to conflicts is a current and growing issue. For example, in 2016 some 75,000 unaccompanied children sought for asylum worldwide^61^, which highlights the importance of studying the effects of this type of separation.

An important finding was that the accumulation of ELS was associated with higher late-life anxiety symptoms. We found that the accumulation of just two stressors significantly increased the odds for clinically significant anxiety. The co-occurrence of stressors may thus further increase the late-life anxiety risk. One potential mechanism for this is that exposure to stressful events early in life may modify brain structures that enhance reactivity to new potentially stressful life events^62^. Our finding corresponds to other studies suggesting that the negative psychological consequences of the accumulation of ELS may carry until late adulthood^27,28^. It is essential to minimize the risk of ELS accumulation by directing early interventions to those already exposed to childhood stressors.

Since our study participants had experienced World War II in childhood, the risk for stressful childhood experiences such as parental loss or separation was relatively high. During World War II, Finland fought two wars characterized by bombardings, food regulations, and deaths of relatives or friends^18,21,47^. An earlier study found that reporting adverse experiences during World War II was associated with an increased risk of anxiety disorders evident still in old age^63^. Still, it is important to note that emotional and physical traumas, low childhood SES and parental divorce were associated with anxiety symptoms even in our sample of those exposed to childhood war-time conditions. After the war, nutrition in Finland was limited and food regulations continued for several years, family sizes were large and infant mortality rate was high^64–66^. On the other hand, the life course of our participants is characterized by Finland undergoing a great structural change and developing to a welfare state during their lifespan. This was indicated by general rise in life expectancy, SES, and level of education in Finland since 1945^64–66^, following a global trend^67^. However, as seen in our study, the SES of the participants remained relatively stable throughout childhood and adulthood. The overall anxiety level in our sample was similar to that found in an earlier study in an elderly community-dwelling population that had also experienced World War II^68^.

Several epigenetic, genetic, physiological, and psychological developmental pathways may contribute to the association between ELS and anxiety. Particularly during early development, chronic or repeated stress exposure may alter the development of brain structures related to stress vulnerability, including hypothalamus-pituitary-adrenal (HPA) axis, amygdala, hippocampus and frontal cortex^29,69–71^. More specifically, chronic childhood stress has been associated with dysregulation of the negative feedback system of the HPA axis^29^, thereby increasing psychopathology risk^70^. Changes in the aforementioned brain structures have been consistently found in mental disorders, including anxiety disorders^72,73^. On a cellular level, the effects of ELS on psychopathology risk may be epigenetically mediated: ELS exposure has been repeatedly linked with increased methylation of the glucocorticoid receptor gene *NR3C1*^30,74^ regulating the glucocorticoid functioning of the HPA axis^30^. Hence, epigenetic changes in the neurobiological stress system as a consequence of ELS may underlie our findings.

This study has several strengths. First, our large and representative study sample allows examining subclinical anxiety symptoms which are common in late adulthood. Secondly, we have broadly studied several forms of ELS, which enables comparing different types of stressors to further clarify the association between ELS and late-life anxiety. Third, the data on childhood separation and SES are register-based and objectively collected. In contrast, data on physical and emotional trauma, parental divorce, and death of a family member are based on retrospective self-report, which disallows making causal interpretations. The partly retrospective data is one limitation of our study, since it may inflate the risk of recall bias due to long recall time. However, it has been suggested that retrospective self-report is associated with under-reporting, rather than over-reporting, past traumatic events^75^. While there might be a reporting bias due to retrospective reporting of ELS, research evidence suggests that people are relatively reliable on their recall of ELS and that reports of ELS are fairly consistent across time^76,77^. It has also been assessed that current mood, e.g. depressive symptoms, have a relatively low effect on the reliability and consistency of self-reported childhood experiences^76^.

Another limitation is that anxiety symptoms are reported at one time point, and data on the onset and persistence of anxiety symptoms could not be obtained. Hence, we cannot specify whether anxiety symptoms have been persistent throughout the life course or are only manifested later in life. For example, in a prospective study, Clark *et al*. noted that the association between ELS and mid-life psychopathology may be partly explained by anxiety in early adulthood^78^. This could not be examined in our data. A third limitation is that, as shown in Table 3, types of ELS often co-occurred and were inter-correlated. This should be taken into account when interpreting our results, as it may have had an effect on our ability to detect independent effects of the different types of ELS. Our findings need to be replicated in even larger samples. Fourth, it is possible that the participants’ experiences hereby defined as ELS, e.g. separation from parents or low parental occupational status, vary highly and are not necessarily regarded as negative. Fifth, the TEC questionnaire was not sent to all participants of the study, and thus our sample size varied in the analyses regarding different ELS types (Table 1). Finally, there was some attrition in the study sample, particularly regarding self-reported traumatic experiences. An earlier study has suggested that participants with severe psychopathology are more likely to drop out in follow up studies^79^. Due to this issue, while our large sample is likely to capture population-level variation in psychopathology, the generalizability of the findings to more severe forms of psychopathology may be limited.

Identifying anxiety symptoms and ELS as their risk factor, as early in life as possible, may substantially promote the well-being of elderly adults, at best by preventing the harmful effects of chronic stress on development with early interventions^80,81^. Acknowledging the potentially life-long negative effects of ELS on brain development and stress reactivity^30,31,70,71^ further highlights the importance of directing early interventions to those exposed to ELS. Also, there are promising results on recognizing the role of early life adversity in psychotherapeutic interventions among elderly people^82^. Screening for potentially stressful childhood experiences in elderly populations may hence work as an important factor in the prevention and treatment of late-life anxiety symptoms.

In conclusion, in our large cohort study, emotional and physical trauma, low childhood SES, and parental divorce were associated with dimensional and/or clinically significant anxiety symptoms in late adulthood, and these associations were mostly independent of each other. Furthermore, the risk of late adulthood anxiety symptoms increased with the accumulation of ELS. This is one of the first studies comparing several different types of potentially stressful childhood experiences and indicating that ELS and its accumulation may be associated with anxiety symptoms even in late adulthood.

## Supplementary information

Supplementary tables S1-S3

## Data Availability

The datasets generated and analysed during the current study are not publicly available due to restrictions on data publicity implemented to ensure the protection of privacy of the participants and the compliance with relevant Finnish laws. The researchers using the data are required to follow the terms of a number of clauses complying with relevant laws designed to ensure the protection of privacy. Data are available from the corresponding author on reasonable request, after obtaining approval from the Steering Committee of the Helsinki Birth Cohort Study. Data requests may be subject to further review by the national register authority National Institute for Health and Welfare.
