# Supplementary material for: The Impact of Early Life Stress on Anxiety Symptoms in Late Adulthood
Source: Sci Rep. 2019 Mar 13;9:4395. doi: 10.1038/s41598-019-40698-0 (PMC6416302; doi:10.1038/s41598-019-40698-0)
Supplement: Supplementary file 1 — Supplementary tables S1-S3 [file 41598_2019_40698_MOESM1_ESM.pdf]

SUPPLEMENTARY INFORMATION

THE IMPACT OF EARLY LIFE STRESS ON ANXIETY SYMPTOMS IN LATE ADULTHOOD

Anna Lähdepuro[a]*, Katri Savolainen[a], Marius Lahti-Pulkkinen[a,b,d], Johan G. Eriksson[c-e], Jari Lahti[a], Soile Tuovinen[a], Eero Kajantie[d,f,g], Anu-Katriina Pesonen[a], Kati Heinonen[a], Katri Räikkönen[a]

[a] Department of Psychology and Logopedics, Faculty of Medicine, University of Helsinki, Finland

[b] Queen's Medical Research Institute, University of Edinburgh, United Kingdom

[c] Department of General Practice and Primary Health Care, University of Helsinki and Helsinki University Hospital, Helsinki, Finland

[d] Chronic Disease Prevention Unit, Department of Public Health Solutions, National Institute for Health and Welfare, Helsinki, Finland

[e] Folkhälsan Research Center, Helsinki, Finland

[f] Children's Hospital, Helsinki University Hospital and University of Helsinki, Helsinki, Finland

[g] PEDEGO Research Unit, MRC Oulu, Oulu University Hospital and University of Oulu, Oulu, Finland

*Corresponding author: Anna Lähdepuro, University of Helsinki, PO Box 21 (Haartmaninkatu 3), 00014 University of Helsinki, Finland; telephone: +358407178812; email: anna.lahdepuro@helsinki.fi.

Supplementary table S1. Linear regression analyses of the association of the age at first potentially traumatic event in childhood with anxiety symptoms in late adulthood.

	N	B	95%CI		p
Age at first physical trauma					
Model 1[a)]	278	0.02	-0.01	0.05	.24
Model 2[b)]	278	0.03	-0.003	0.06	.08
Age at first emotional trauma					
Model 1[a)]	299	-0.02	-0.04	0.01	.19
Model 2[b)]	299	-0.02	-0.04	0.01	.28
Age at separation					
Model 1[a)]	194	-0.01	-0.14	0.12	.89
Model 2[b)]	194	-0.02	-0.16	0.12	.77
Age at parental divorce					
Model 1[a)]	97	0.03	-0.02	0.08	.27
Model 2[b)]	97	0.03	-0.02	0.08	.27
Age at death of a family member					
Model 1[a)]	186	0.001	-0.03	0.03	.96
Model 2[b)]	186	0.002	-0.03	0.03	.91

a) The studied stressful experience is included in the model as an independent variable, while age and sex are used as covariates. For physical trauma, emotional trauma, parental divorce and death of a family member, also the interval between TEC and BAI was used as a covariate.
b) The studied stressful experience is included in the model as an independent variable with other stressful experiences, while age and sex are used as covariates. For physical trauma, emotional trauma, parental divorce and death of a family member, also the interval between TEC and BAI was used as a covariate.

Supplementary table S2. Linear regression analyses of the association of potentially stressful events in childhood with anxiety symptoms in late adulthood after excluding participants with missing values on the BAI from the analyses.

	N	B	95%CI		p
Self-reported physical trauma in childhood(yes/no)					
Model 1[a)]	1006	0.30	0.17	0.44	<.001
Model 2[b)]	1006	0.15	-0.01	0.30	.07
Self-reported emotional trauma in childhood (yes/no)					
Model 1[a)]	988	0.42	0.29	0.56	<.001
Model 2[b)]	988	0.36	0.20	0.52	<.001
Childhood SES (manual worker/clerical worker)					
Model 1[a)]	1802	0.09	-0.002	0.18	.06
Model 2[b)]	1802	0.11	0.02	0.20	.02
Separation from parents					
Evacuee - Model 1[a)]	1803	0.05	-0.10	0.20	.51
Evacuee - Model 2[b)]	1802	-0.01	-0.16	0.14	.92
Self-reported separation - Model 1[a)]	1803	0.13	-0.05	0.31	.16
Self-reported separation - Model 2[b)]	1802	0.08	-0.11	0.26	.41
Parental divorce					
Model 1[a)]	1172	0.13	-0.08	0.34	.22
Model 2[b)]	1172	0.08	-0.13	0.28	.47
Death of a family member					
Model 1[a)]	1154	0.07	-0.09	0.23	.39
Model 2[b)]	1154	0.05	-0.11	0.21	.53
Accumulation of risk factors[c)]	1222				
1 stressful event		0.19	0.05	0.33	.01
2 stressful events		0.34	0.18	0.49	<.001
3 or more stressful events		0.42	0.24	0.60	<.001

a) The studied stressful experience was included in the model as an independent variable, while age and sex were used as covariates. For physical trauma, emotional trauma, parental divorce and death of a family member, also the interval between TEC and BAI was used as a covariate.

b) The studied stressful experience was included in the model as an independent variable with other stressful experiences, while age and sex were used as covariates. For physical trauma, emotional trauma, parental divorce and death of a family member, also the interval between TEC and BAI was used as a covariate.

c)The person has experienced 0, 1, 2, or 3 or more different ELS types in childhood. The group with 0 stressful events in childhood functions as a comparison group.

Supplementary table S3. Logistic regression analyses of the association between potentially stressful events in childhood and the odds ratio of clinically significant anxiety symptoms in late adulthood after excluding participants with missing values on the BAI from the analyses.

	N	OR	95%CI		p
Self-reported physical trauma in childhood(yes/no)	1006				
Model 1[a)]		1.56	0.97	2.52	.07
Model 2[b)]		0.92	0.52	1.61	.76
Self-reported emotional trauma in childhood (yes/no)	988				
Model 1[a)]		2.61	1.65	4.12	<.001
Model 2[b)]		2.62	1.52	4.49	<.001
Childhood SES (manual worker/clerical worker)	1802				
Model 1[a)]		1.28	0.91	1.82	.16
Model 2[b)]		1.33	0.93	1.90	.11
Separation from parents					
Evacuee - Model 1[a)]	1803	1.36	0.81	2.27	.25
Evacuee - Model 2[b)]	1802	1.17	0.69	1.98	.56
Self-reported separation - Model 1[a)]	1803	1.26	0.69	2.29	.45
Self-reported separation - Model 2[b)]	1802	1.05	0.57	1.95	.87
Parental divorce	1172				
Model 1[a)]		2.35	1.31	4.24	.004
Model 2[b)]		2.16	1.17	3.99	.01
Death of a family member	1154				
Model 1[a)]		1.05	0.61	1.82	.86
Model 2[b)]		1.00	0.57	1.76	1.00
Accumulation of risk factors[c)]	1222				
1 stressful event		1.41	0.78	2.56	.27
2 stressful events		1.87	1.00	3.48	.05
3 or more stressful events		2.59	1.36	4.94	.004

a) The studied stressful experience was included in the model as an independent variable, while age and sex were used as covariates. For physical trauma, emotional trauma, parental divorce and death of a family member, also the interval between TEC and BAI was used as a covariate.
b) The studied stressful experience was included in the model as an independent variable with other stressful experiences, while age and sex were used as covariates. For physical trauma, emotional trauma, parental divorce and death of a family member, also the interval between TEC and BAI was used as a covariate.

c) The person has experienced 0, 1, 2, or 3 or more stressful events in childhood. The group with 0 stressful events in childhood functions as a comparison group.
